# Beam angle comparison for distal esophageal carcinoma patients treated with intensity‐modulated proton therapy

**DOI:** 10.1002/acm2.13049

**Published:** 2020-10-15

**Authors:** Hongying Feng, Terence T. Sio, William G. Rule, Ronik S. Bhangoo, Pedro Lara, Christopher L. Patrick, Shawn Korte, Mirek Fatyga, William W. Wong, Steven E. Schild, Jonathan B. Ashman, Wei Liu

**Affiliations:** ^1^ Department of Radiation Oncology Mayo Clinic Phoenix AZ USA

**Keywords:** beam angle, esophageal cancer, intensity‐modulated proton therapy, interplay effect

## Abstract

**Purpose:**

To compare the dosimetric performances of intensity‐modulated proton therapy (IMPT) plans generated with two different beam angle configurations (the Right–Left oblique posterior beams and the Superior–Inferior oblique posterior beams) for the treatment of distal esophageal carcinoma in the presence of uncertainties and interplay effect.

**Methods and Materials:**

Twenty patients’ IMPT plans were retrospectively selected, with 10 patients treated with the R‐L oblique posterior beams (Group R‐L) and the other 10 patients treated with the S‐I oblique posterior beams (Group S‐I). Patients in both groups were matched by their clinical target volumes (CTVs—high and low dose levels) and respiratory motion amplitudes. Dose‐volume‐histogram (DVH) indices were used to assess plan quality. DVH bandwidth was calculated to evaluate plan robustness. Interplay effect was quantified using four‐dimensional (4D) dynamic dose calculation with random respiratory starting phase of each fraction. Normal tissue complication probability (NTCP) for heart, liver, and lung was calculated, respectively, to estimate the clinical outcomes. Wilcoxon signed‐rank test was used for statistical comparison between the two groups.

**Results:**

Compared with plans in Group R‐L, plans in Group S‐I resulted in significantly lower liver D_mean_ and lung V_30Gy[RBE]_ with slightly higher but clinically acceptable spinal cord D_max_. Similar plan robustness was observed between the two groups. When interplay effect was considered, plans in Group S‐I performed statistically better for heart D_mean_ and V_30Gy[RBE]_, lung Dmean and V_5Gy[RBE]_, and liver D_mean_, with slightly increased but clinically acceptable spinal cord D_max_. NTCP for liver was significantly better in Group S‐I.

**Conclusions:**

IMPT plans in Group S‐I have better sparing of liver, heart, and lungs at the slight cost of spinal cord maximum dose protection, and are more interplay‐effect resilient compared to IMPT plans in Group R‐L. Our study supports the routine use of the S‐I oblique posterior beams for the treatments of distal esophageal carcinoma.

## 1 INTRODUCTION

Esophageal carcinoma is the eighth most common malignancy and the sixth most common cause of cancer death worldwide.^1^ In the United States, the 5‐yr relative survival rate for the esophageal cancer patients is low, only about 20%.^2^ Due to the proximity of organs‐at‐risk for distal esophageal cancer patients, the delivery of tumoricidal dose of radiation without causing significant toxicity is challenging.^3^, ^4^

Intensity‐modulated proton therapy (IMPT) has distinct advantages in terms of high conformality of target coverage and organs at risk (OARs) protection^5^, ^6^, ^7^, ^8^. However, compared to photon‐based intensity‐modulated radiation therapy (IMRT) and the older passive‐scattering proton therapy (PSPT), IMPT can suffer from greater sensitivity to proton beam range and patient setup uncertainties.^9^, ^10^, ^11^ Additionally, respiratory motion makes IMPT even more vulnerable because of the interplay effect, which is caused by the interference between dynamic beamlet delivery and intra‐fractional tumor motion.^12^, ^13^, ^14^, ^15^, ^16^, ^17^, ^18^, ^19^, ^20^, ^21^, ^22^, ^23^ This poses a greater challenge when treating distal esophageal carcinoma due to adjacent diaphragmatic and gastric motion.

Robust optimization^24^, ^25^, ^26^, ^27^, ^28^, ^29^, ^30^, ^31^, ^32^, ^33^, ^34^, ^35^, ^36^, ^37^, ^38^, ^39^, ^40^, ^41^, ^42^, ^43^, ^44^, ^45^, ^46^, ^47^ has been introduced to mitigate the impact of uncertainties, and is now widely accepted in the routine proton clinical practice. However, techniques to mitigate interplay effect would still require further improvement.^48^ Abdominal compression and breath holding can help limit the motion, but often result in less patient comfort during treatments. Gating^49^ and repainting^13^, ^16^, ^50^ can provide better dose distribution during motion, but will inherently prolong the treatment time. Tumor tracking^51^, ^52^ and the recently proposed 4D optimization^45^, ^53^, ^54^, ^55^ are still in development, and will need further verifications prior to routine clinical use. Currently at our institution, IMPT plans are derived for distal esophageal cancer based on the averaged four‐dimensional computed tomography (4D‐CT) with uncertainties considered. Generated IMPT plans are then verified by plan evaluation on maximum exhalation and maximum inhalation respiratory phases, as well as a separate interplay effect calculation.^45^

Previous dosimetric studies^56^, ^57^, ^58^, ^59^ of esophageal carcinoma treatment compared both proton and photon treatments. Zhang et al. reported that proton therapy provided significantly better sparing of the lungs than IMRT.^56^ Shiraishi et al. found that in patients with mid to distal esophageal cancer, proton therapy resulted in significantly lower radiation exposure to the whole heart and cardiac structures compared to IMRT.^57^ Lin et al. concluded in a large‐scale multi‐institutional study that proton therapy was associated with significantly less postoperative complications and shorter length of in‐hospital stay than 3D conformal radiation therapy and IMRT.^58^ Liu et al. carried out a comparative study between small‐spot IMPT and volumetric‐modulated arc therapy (VMAT), and found that small‐spot IMPT significantly improved sparing of heart, liver, and lungs with clinically acceptable plan robustness.^34^ Most recently, Lin et al. reported the result of a phase IIB trial that for locally advanced esophageal cancer, proton beam therapy (PBT) reduced the risk and severity of adverse events (AEs) while maintaining similar progression‐free survival (PFS) when compared with IMRT.^59^

**Fig. 1:**
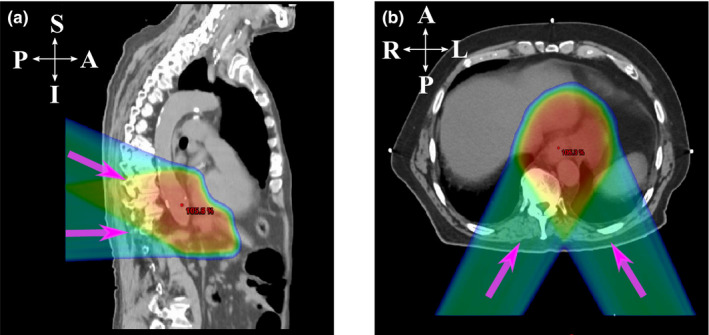
Two different beam angle configurations. The purple arrows indicate different beam directions. (a) Sagittal plane of a typical patient treated with Superior–Inferior (S‐I) oblique posterior beams, and (b) transverse plane of a typical patient treated with Right–Left (R‐L) oblique posterior beams.

Specifically for IMPT, different configurations may lead to different outcomes, such as spot sizing and spacing.^38^ The selection of beam angles is important as well. At our institution, two different sets of beam angles are typically used for IMPT planning for distal esophageal cancer: the Right–Left (R‐L) oblique posterior beams (Group R‐L) and the Superior–Inferior (S‐I) oblique posterior beams (Group S‐I) (Fig. 1). To the best of our knowledge, no comparative study has been reported comparing these two different configurations of beam angles in IMPT for the treatment of distal esophageal cancer. In this study, we compared the IMPT plan quality and robustness for distal esophageal carcinoma between R‐L oblique posterior beams (Group R‐L) and S‐I oblique posterior beams (Group S‐I). The interplay effect was also quantified for both beam orientations.

## 2 MATERIALS AND METHODS

### 2.A Patient selection

Twenty patients with distal esophageal carcinoma treated with IMPT at our institution between March 2017 and December 2019 were retrospectively reviewed. All these patients were treated with the same prescription doses for the clinical target volumes (CTVs) (50 Gy[RBE] for CTV_high_ and 45 Gy[RBE] for CTV_low_). Prescription doses for both volumes (CTV_high_ and CTV_low_) were typically delivered in 25 daily fractions via simultaneously integrated boost (SIB).

To assess the impact of different beam angles, patients were chosen based on the dosimetrist’s choice for beam angles at the time of IMPT planning, typically two sets, thus could be divided into two groups. In the first group, two oblique posterior beams in the S‐I direction with a couch angle of 270° were employed (Group S‐I). In the second group, two oblique posterior beams in the R‐L direction of a couch angle of 180° were employed (Group R‐L). In Group R‐L, a few patients were treated with one or two additional anterior beams for better target dose distribution and adjacent organ protection (See Table S1).

For all patients, tumor locations were distal (ie, near the gastroesophageal junction), where the impact of the respiratory motion was important and the protection of OARs could be difficult anatomically. In our earlier proton practice, the patients were planned and treated with mainly oblique posterior beams in the R‐L direction. We then started exploring using oblique posterior beams in the S‐I directions, which has become more commonly used recently. Thus, 20 patients (10 in each group) were selected for this comparison. We matched the patients by comparing their CTV volumes and also tumor motion amplitudes, so that they were most presentative clinically for statistical comparison (Table 1). Tumor motion amplitude in this paper is defined as the largest motion in one direction (S‐I, A‐P, or R‐L, see Table S2). Additionally, no patients had any implanted electronic devices, and no range shifter was used in the treatment of any patients. All selected patients were treated with curative intent as determined by the clinical radiation oncologists.

**Table 1:** Target characteristics between two treatment/beam angle groups.

	Group S‐I	Group R‐L	*P*‐value^a^
Patient number	10	10	
CTV_high_ (cm^3^) [median (range)]	179.4 (132.2 ~ 295.6)	207.4 (56.8 ~ 338.6)	0.285
CTV_low_ (cm^3^) [median(range)]	390.5 (242.6 ~ 546.3)	415.8 (279.0 ~ 732.9)	0.879
Tumor motion amplitude (cm) [median (range)]	0.8 (0.5 ~ 0.9)	0.8 (0.6 ~ 1.0)	0.261

^a^ The Wilcoxon signed‐rank test was used.

### 2.B Treatment planning

Treatment planning was carried out for all patients by using the commercial treatment planning system Eclipse^TM^ (version 13, Varian Medical System, Palo Alto, CA). All the IMPT plans were generated on the averaged 4D‐CT with CTV density override (HU = 50) for improving plan robustness related to respiratory motion. An optimization target volume (OTV) was constructed by adding a uniform 5‐mm margin to the CTV to assist the robust plan generation. At least one spot was placed outside OTV to ensure the forming of a uniform dose distribution within OTV. Discrete energy layers from 71.3 to 205.3 MeV were used (Table S1).

For beam placement, two posterior beams were typically used, whereas one or two additional anterior beams could be optionally introduced as needed to achieve a clinically acceptable plan (ie, meeting the institutional dose‐volume constraints and plan robustness requirements). More details regarding treatment planning for distal esophageal carcinoma can be found in Liu et al.^34^ For the current study, as described in Section 2.A, all patients from Group S‐I had two S‐I oblique posterior beams with no additional supplemental beams required. For the 10 patients from the Group R‐L, besides two R‐L oblique posterior beams, two patients had one extra anterior beam each and one patient had two additional anterior beams (Table S1).

Pencil‐beam convolution supposition (PCS) was used for all patients to carry out the volume‐dose calculation and beamline modifiers. For optimization, single‐field optimization (SFO) was usually applied as the preferred method. Alternative multiple‐field optimization (MFO) would be used if SFO failed to meet the clinical requirements at first try. For the SFO approach, the PCS model was utilized; for the MFO approach, the nonlinear uniform proton optimizer (NUPO) was used. When MFO approach was used, an evaluation of dose distribution per field would be done using Eclipse^TM^ to make sure the per field gradient was shallow, so that the plan would be robust to independent beam shifts during the delivery. In all selected patients, the plan optimizations of six patients used SFO method, with three patients in each group. After the planning on the averaged 4D‐CT, two verification plans on the maximum inhalation and maximum exhalation phases without the density override were generated to evaluate the influence of respiratory motion, which in turn acted as guidance for the adjustment of the original plan generated on the averaged 4D‐CT. When all three plans (the original plan on the averaged 4D‐CT and two verification plans on the maximum inhalation and exhalation phases) had met the clinical requirements (Table 2), they could be considered optimal.

**Table 2:** Dose‐volume constraints for organs‐at‐risk.

Structure	Dose limits^a^
Liver	D_mean_ < 25 Gy[RBE]; V_30Gy[RBE]_ < 60%
Total lung	V_5Gy[RBE]_ < 60%; V_20Gy[RBE]_ < 15%; D_mean_ < 15 Gy[RBE]
Spinal cord	D_max_ < 45 Gy[RBE]; V_45Gy[RBE]_ < 0.1%
Heart	V_25Gy[RBE]_ <50%; V_40Gy[RBE]_ < 30%; D_mean_ < 26 Gy[RBE]
Left/right kidney	V_18Gy[RBE]_ < 10%; D_mean_ < 18 Gy[RBE]

^a^ These DVCs are carefully generated by experienced radiation oncologists and physicists at our institution. They are generally more restrictive (thus safer) than the ones recommended by RTOG.

### 2.C Treatment delivery

Hitachi ProBeat‐V spot‐scanning proton machines (Hitachi, Tokyo, Japan) were used to deliver the treatment at our institution. The energy range of the machine was from 71.3 to 228.8 MeV with 97 discrete energy layers. The characteristic times during the delivery were: approximately 1 s in proton acceleration or deceleration, averaged 1.91 s in energy switching, averaged 1.93 ms in magnet preparation and verification, averaged 7.9 s in proton extraction, approximately 0.1 s in extraction setup, and approximately 3 ms of time interval between spots. The spot dose delivery rate is 8.7 MU/s.^60^

### 2.D Plan quality evaluation

For the target volume, we calculated the D_95%_ (the minimum dose covering the lowest 95% of the irradiated structure’s volume), D_5%_ (the minimum dose covering the highest irradiated 5% of the structure’s volume), and D_2cc_ (the minimum dose covering the highest irradiated 2cc of the structure’s volume) of both CTV_high_ and CTV_low_ according to the corresponding dose‐volume‐histograms (DVHs). Each of the CTV‐related parameters was normalized by the corresponding prescribed dose (CTV_high_ by 50 Gy [RBE] and CTV_low_ by 45 Gy [RBE]). CTV D95%, D5%‐D95%, and D_2cc_ were used to illustrate target dose coverage, target dose homogeneity, and hot spots, respectively.

For the OAR protection, we calculated lung D_mean_ (mean dose), spinal cord D_max_ (maximum dose), heart D_mean_, and liver D_mean_. In addition, we also calculated relative volumes V_5Gy[RBE]_ and V_20Gy[RBE]_ for lung, V_30Gy[RBE]_ and V_40Gy[RBE]_ for heart, V_30Gy[RBE]_ for liver, V_18Gy[RBE]_ for kidney, and V_45Gy[RBE]cc_ for stomach, with V_XGy[RBE]_ and V_XGy[RBE]cc_ defined as the normalized and absolute (cc) volume of a structure receiving at least X Gy[RBE] dose, respectively.

### 2.E Robustness qualification

Patient setup uncertainties were considered by a 3‐mm rigid shift in both positive and negative directions along the three axes of anterior–posterior (A‐P), right‐left (R‐L), and superior–inferior (S‐I). As the contouring of the target volumes (CTV_high_ and CTV_low_) had already taken the anatomical constraints, the pathology and location of the tumor, and the potential microscopic tumor extent and anatomic boundaries of heart, lungs, liver, kidneys, and bowel,^34^ a 3‐mm rigid shift in all six directions was considered appropriate for the patient setup uncertainties. Patient range uncertainties were considered by scaling the nominal beam range up and down by 3%. Altogether, a set of 13 different scenarios was generated (one nominal and 12 perturbed scenarios). A band graph of DVHs corresponding to all 13 scenarios was obtained. The bandwidth was then used to evaluate the plan robustness, with the horizontal bandwidth for the dose at a reference volume and the vertical bandwidth for the volume at a reference dose. Smaller bandwidth would indicate better plan robustness.

### 2.F Interplay effect evaluation

Dose calculation software was developed at our institution to assess dose distribution in the presence of interplay effect. Every spot for each field per fraction was assigned to the corresponding respiratory phase according to their temporal relationship with the spot delivery sequence and patient‐specific respiratory motions. Then, the dose of each phase was calculated with respect to the assigned spots. Finally, the calculated dose of each phase was then deformed onto the reference phase (maximum exhalation phase) through deformable image registration to get the final 4D dynamic dose.^45^ Our software can handle 10 respiratory phases; however, in practice, only two equally weighted extreme phases (maximum exhalation and maximum inhalation phases) would be selected due to computation time considerations. The starting phase for each field per fraction was randomized to help mitigate its influence. The actual fraction number of the treatment plan was used in the calculation. The multiple energy extraction (MEE) method was adopted for more efficient delivery compared to the single energy extraction (SEE) method.^61^, ^62^

Iso‐layer repainting was utilized to mitigate the impact of interplay effect. During the repainting process, a minimum MU limit of 0.003 MU and a patient‐specific maximum MU limit of our proton machine were employed. When the respiratory motion amplitude was within 5 mm, the maximum MU limit was set to be 0.04 MU. Otherwise, the maximum MU limit was changed to 0.01 MU for more repainting in order to mitigate interplay effect due to increased respiratory motions. Any MU values larger than the maximum limit were split into several less intensive spots with their intensities equal to the maximum MU limit and one possible residual spot. If the intensity of a spot or a residual spot was between the minimum and maximum MU limits, it was delivered with its actual intensity. For a spot or a residual spot that had intensity less than the minimum MU limit, its delivery was adjusted according to its intensity. If the intensity was larger than half of the minimum limit, it was rounded up to be the minimum MU limit, while otherwise, the spot was dropped. Note that the maximum and minimum MU limits mentioned here were well investigated and benchmarked machine‐specifically only within our institution, thus might only apply well on our machine.

### 2.G Normal tissue complication probability evaluation

To estimate the clinical outcomes of the IMPT plans for patients from each group, the normal tissue complication probability (NTCP) was calculated for heart, lung, and liver for every patient in the nominal scenario on averaged CT. Based on the DVH information, the Niemierko model^63^, ^64^ was used for the NTCP calculation.

### 2.H Statistical analysis

For fair comparison, all plans were normalized to have a CTV_low_ D95% of 100% of the prescribed dose in the nominal scenario (without uncertainties or interplay effect considered). Wilcoxon signed‐rank test was applied to carry out the statistical analysis between the selected paired groups. *P* < 0.05 was considered to be statistically significant. Box‐and‐Whisker plotting was adopted to illustrate the DVH indices for all patients from each group. Any value >1.5 times of the interquartile range above or below the quartile limits was considered as an outlier.

## 3 RESULTS

### 3.A Plan quality in the nominal scenario

**Fig. 2:**
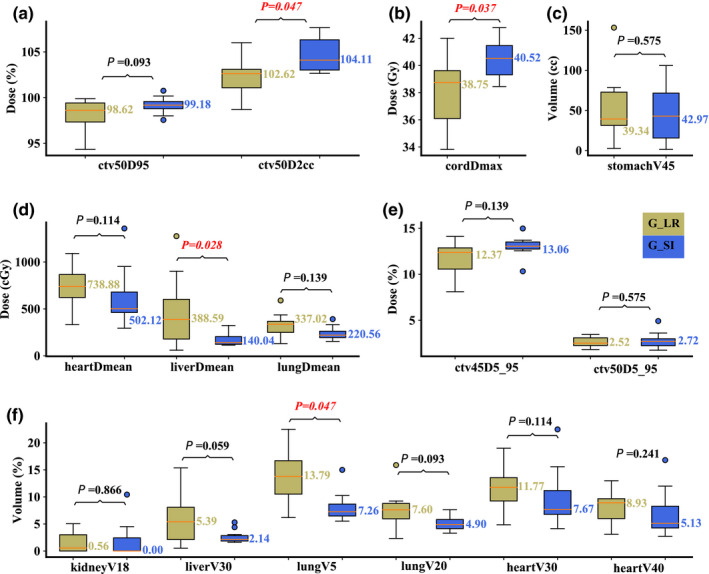
Comparison of the dose‐volume‐histogram indices between plans of Group S‐I and Group R‐L on averaged 4D‐CT. (a) Normalized CTV_high_ D_95%_ and CTV_high_ D_2cc_, (b) spinal cord D_max_, (c) stomach V_45Gy[RBE]cc_, (d) heart D_mean_, liver D_mean_, and lung D_mean_, (e) normalized CTV_low_ D_5%_‐D_95%_ and CTV_high_ D_5%_‐D_95%_, and (f) normalized volume of kidney V_18Gy[RBE]_, liver V_30Gy[RBE]_, lung V_5Gy[RBE]_, lung V_20Gy[RBE]_, heart V_30Gy[RBE]_, and heart V_40Gy[RBE]_. Boxes in khaki are the results from Group R‐L, while blue boxes are the results from Group S‐I. Numbers on the top are *P*‐values from Wilcoxon signed‐rank test. *P*‐values that indicate statistical significance (<0.05) are in red and italicized.

On the averaged 4D‐CT in the nominal scenario, compared to Group R‐L, Group S‐I had significantly better dosimetric values in terms of liver D_mean_ (median value: 140.04 vs 388.59 cGy; *P* = 0.028; the latter value refers to “Group R‐L” here and also hereafter) and total lung V_5Gy[RBE]_ (median value: 7.26 vs 13.79%; *P* = 0.047), but resulted in significantly worse hot spot control CTV_high_ D_2cc_ (median value: 102.62 vs 104.11%; *P* = 0.047) and spinal cord D_max_ (median value: 40.52 vs 38.75 Gy; *P* = 0.037). Other DVH indices were comparable [Figs. 2(a)–2(f)].

**Fig. 3:**
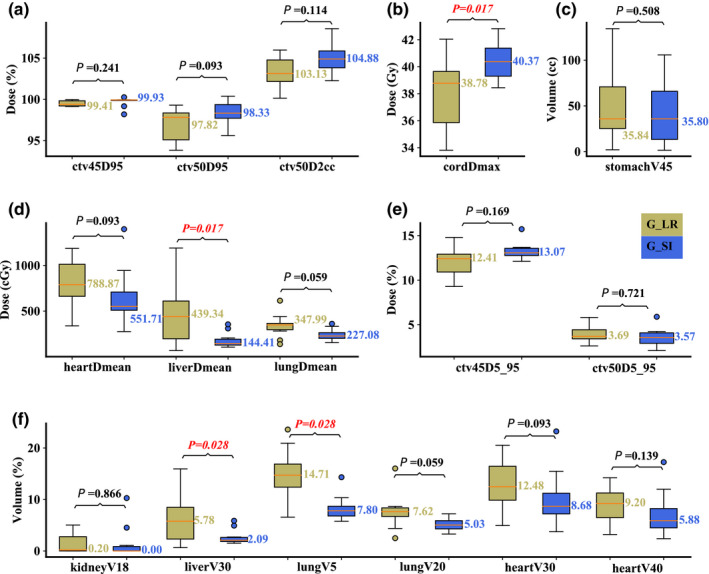
Comparison of the dose‐volume‐histogram indices between plans of Group S‐I and Group R‐L on the maximum inhalation phase. (a) Normalized CTV_high_ D_95%_ and CTV_high_ D_2cc_, (b) spinal cord D_max_, (c) stomach V_45Gy[RBE]cc_, (d) heart D_mean_, liver D_mean_, and lung D_mean_, (e) normalized CTV_low_ D_5%_‐D_95%_ and CTV_high_ D_5%_‐D_95%_, and (f) normalized volume of kidney V_18Gy[RBE]_, liver V_30Gy[RBE]_, lung V_5Gy[RBE]_, lung V_20Gy[RBE]_, heart V_30Gy[RBE]_, and heart V_40Gy[RBE]_. Boxes in khaki are the results from Group R‐L, while blue boxes are the results from Group S‐I. Numbers on the top are *P*‐values from Wilcoxon signed‐rank test. *P*‐values that indicate statistical significance (<0.05) are in red and italicized.

**Fig. 4:**
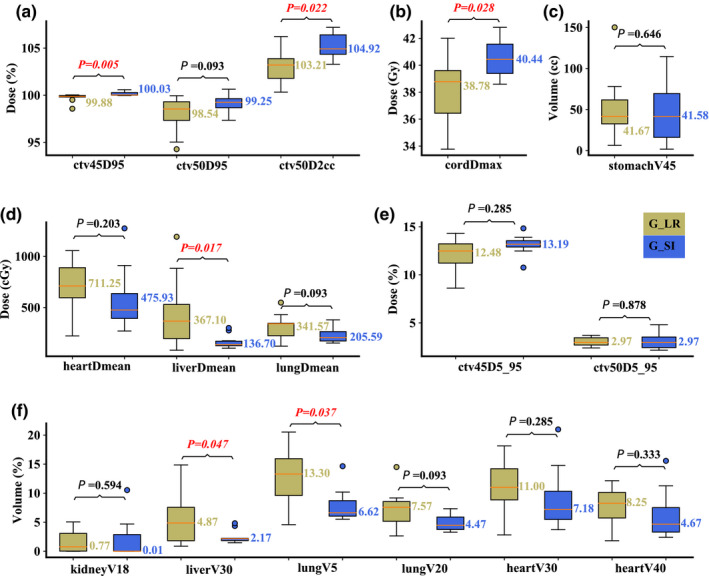
Comparison of the dose‐volume‐histogram indices between plans of Group S‐I and Group R‐L on the maximum exhalation phase. (a) Normalized CTV_high_ D_95%_ and CTV_high_ D_2cc_, (b) spinal cord D_max_, (c) stomach V_45Gy[RBE]cc_, (d) heart D_mean_, liver D_mean_, and lung D_mean_, (e) normalized CTV_low_ D_5%_‐D_95%_ and CTV_high_ D_5%_‐D_95%_, and (f) normalized volume of kidney V_18Gy[RBE]_, liver V_30Gy[RBE]_, lung V_5Gy[RBE]_, lung V_20Gy[RBE]_, heart V_30Gy[RBE]_, and heart V_40Gy[RBE]_. Boxes in khaki are the results from Group R‐L, while blue boxes are the results from Group S‐I. Numbers on the top are *P*‐values from Wilcoxon signed‐rank test. *P*‐values that indicate statistical significance (<0.05) are in red and italicized.

On the maximum inhalation phase in the nominal scenario, compared to Group R‐L, Group S‐I performed significantly better in liver D_mean_ (median value: 144.41 vs 439.34 cGy; *P* = 0.017) and V_30Gy[RBE]_ (median value: 2.09 vs 5.78%; *P* = 0.028), and lung V_5Gy[RBE]_ (median value: 7.80 vs 14.71%; *P* = 0.028), but significantly worse in spinal cord D_max_ (median value: 40.37 vs 38.78 Gy; *P* = 0.017). All other DVH indices were comparable [Figs. 3(a)–3(f)]. On the maximum exhalation phase in the nominal scenario, similar results were obtained with liver D_mean_ (median value: 136.7 vs 367.1 cGy; *P* = 0.017) and V_30Gy[RBE]_ (median value: 2.17 vs 4.87%; *P* = 0.047), and lung V_5Gy[RBE]_ (median value: 6.62 vs 13.3%; *P* = 0.037). Group S‐I was also slightly better in CTV_low_ D_95%_ (median value: 100.03 vs 99.88%; *P* = 0.005) in this scenario [Figs. 4(a)–4(f)].

### 3.B Plan robustness

**Fig. 5:**
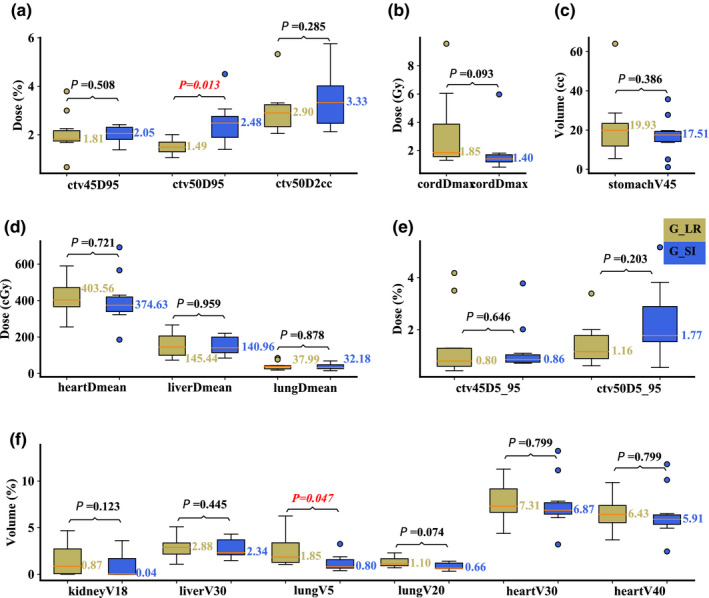
Comparison of the dose‐volume‐histogram bandwidths between plans of Group S‐I and Group R‐L on the averaged 4D‐CT. (a) Normalized CTV_high_ D_95%_ and CTV_high_ D_2cc_, (b) spinal cord D_max_, (c) stomach V_45Gy[RBE]cc_, (d) heart D_mean_, liver D_mean_, and lung D_mean_, (e) normalized CTV_low_ D_5%_‐D_95%_ and CTV_high_ D_5%_‐D_95%_, and (f) normalized volume of kidney V_18Gy[RBE]_, liver V_30Gy[RBE]_, lung V_5Gy[RBE]_, lung V_20Gy[RBE]_, heart V_30Gy[RBE]_, and heart V_40Gy[RBE]_. Boxes in khaki are the results from Group R‐L, while blue boxes are the results from Group S‐I. Numbers on the top are *P*‐values from Wilcoxon signed‐rank test. *P*‐values that indicate statistical significance (<0.05) are in red and italicized.

**Fig. 6:**
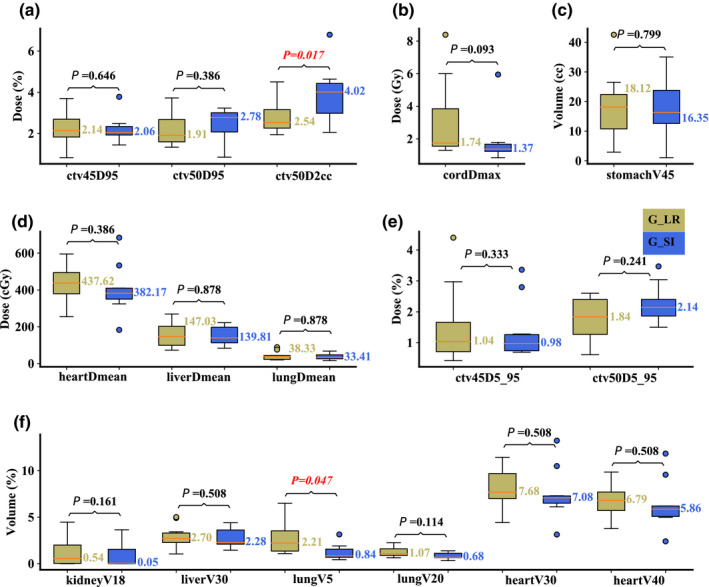
Comparison of the dose‐volume‐histogram bandwidths between plans of Group S‐I and Group R‐L on the maximum inhalation phase. (a) Normalized CTV_high_ D_95%_ and CTV_high_ D_2cc_, (b) spinal cord D_max_, (c) stomach V_45Gy[RBE]cc_, (d) heart D_mean_, liver D_mean_, and lung D_mean_, (e) normalized CTV_low_ D_5%_‐D_95%_ and CTV_high_ D_5%_‐D_95%_, and (f) normalized volume of kidney V_18Gy[RBE]_, liver V_30Gy[RBE]_, lung V_5Gy[RBE]_, lung V_20Gy[RBE]_, heart V_30Gy[RBE]_, and heart V_40Gy[RBE]_. Boxes in khaki are the results from Group R‐L, while blue boxes are the results from Group S‐I. Numbers on the top are *P*‐values from Wilcoxon signed‐rank test. *P*‐values that indicate statistical significance (<0.05) are in red and italicized.

**Fig. 7:**
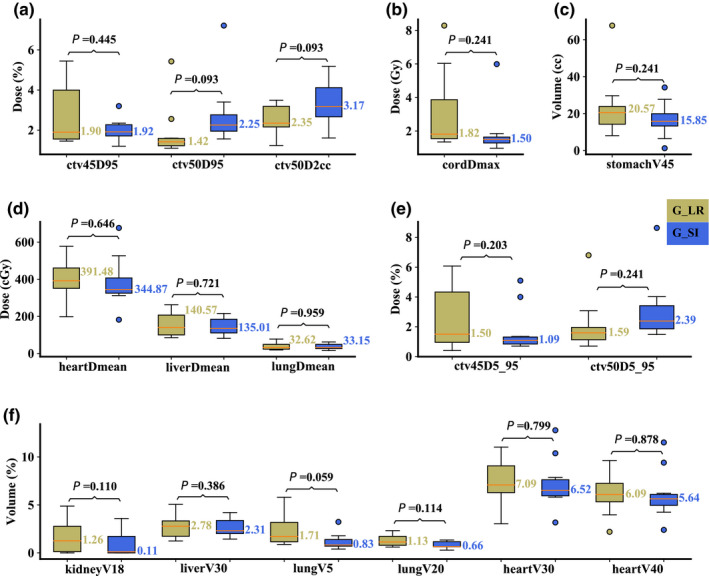
Comparison of the dose‐volume‐histogram bandwidths between plans of Group S‐I and Group R‐L on the maximum exhalation phase. (a) Normalized CTV_high_ D_95%_ and CTV_high_ D_2cc_, (b) spinal cord D_max_, (c) stomach V_45Gy[RBE]cc_, (d) heart D_mean_, liver D_mean_, and lung D_mean_, (e) normalized CTV_low_ D_5%_‐D_95%_ and CTV_high_ D_5%_‐D_95%_, and (f) normalized volume of kidney V_18Gy[RBE]_, liver V_30Gy[RBE]_, lung V_5Gy[RBE]_, lung V_20Gy[RBE]_, heart V_30Gy[RBE]_, and heart V_40Gy[RBE]_. Boxes in khaki are the results from Group R‐L, while blue boxes are the results from Group S‐I. Numbers on the top are *P*‐values from Wilcoxon signed‐rank test. *P*‐values that indicate statistical significance (<0.05) are in red and italicized.

On the averaged 4D‐CT, compared to Group R‐L, Group S‐I had significantly better plan robustness for lung V_5Gy[RBE]_ (median value: 0.80 vs 1.85%; *P* = 0.047), but significantly worse plan robustness for CTV_high_ D_95%_ (median value: 2.48 vs 1.49%; *P* = 0.013). The robustness for other structures was comparable [Figs. 5(a)–5(f)]. On the maximum inhalation phase, significantly better plan robustness of lung V_5Gy[RBE]_ (median value: 0.84 vs 2.21%; *P* = 0.047) in Group S‐I was observed, whereas significantly better plan robustness of CTV_high_ D_2cc_ (median value: 4.02 vs 2.54%; *P* = 0.017) was observed in Group R‐L [Figs. 6(a)–6(f)]. On the maximum exhalation phase, no significant differences for any DVH indices from both groups were observed [Figs. 7(a)–7(f)].

### 3.C Interplay effect

**Fig. 8:**
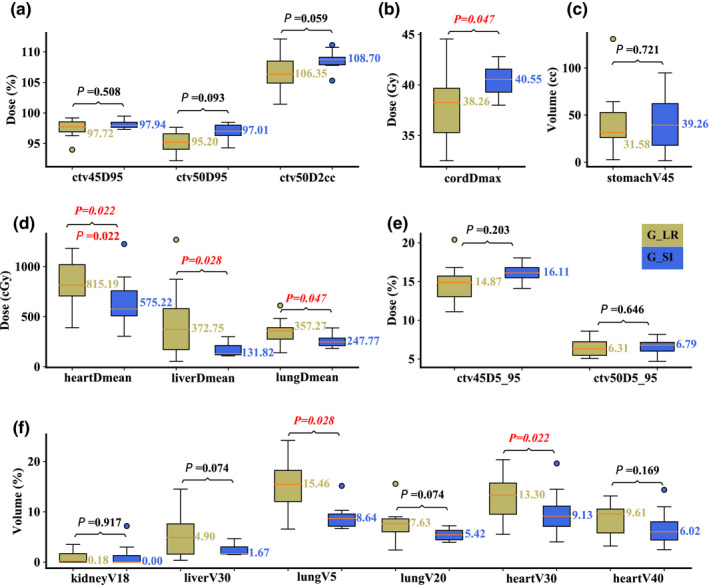
Comparison of the dose‐volume‐histogram indices between plans of Group S‐I and Group R‐L with interplay effect considered. (a) Normalized CTV_high_ D_95%_ and CTV_high_ D_2cc_, (b) spinal cord D_max_, (c) stomach V_45Gy[RBE]cc_, (d) heart D_mean_, liver D_mean_, and lung D_mean_, (e) normalized CTV_low_ D_5%_‐D_95%_ and CTV_high_ D_5%_‐D_95%_, and (f) normalized volume of kidney V_18Gy[RBE]_, liver V_30Gy[RBE]_, lung V_5Gy[RBE]_, lung V_20Gy[RBE]_, heart V_30Gy[RBE]_, and heart V_40Gy[RBE]_. Boxes in khaki are the results from Group R‐L, while blue boxes are the results from Group S‐I. Numbers on the top are *P*‐values from Wilcoxon signed‐rank test. *P*‐values that indicate statistical significance (<0.05) are in red and italicized.

Compared to Group R‐L, Group S‐I resulted in significantly better liver D_mean_ (median value: 575.22 vs 815.19 cGy; *P* = 0.022), heart D_mean_ (median value: 131.82 vs 372.75 cGy; *P* = 0.028), lung D_mean_ (median value: 247.77 vs 357.27 cGy; *P* = 0.047), lung V_5Gy[RBE]_ (median value: 8.64 vs 15.46%; *P* = 0.028), and heart V_30Gy[RBE]_ (median value: 9.13 vs 13.3%; *P* = 0.022). Group R‐L performed significantly better in spinal cord D_max_ (median value: 40.55 vs 38.26 Gy; *P* = 0.047). All other DVH indices were comparable [Figs. 8(a)–8(f)].

### 3.D NTCP

Pericarditis, liver failure, and pneumonitis are considered as the endpoints for heart, liver, and lung, respectively, in the NTCP calculation. Seven of 10 patients from the Group S‐I had lower NTCP for heart, 9 of 10 patients from the Group S‐I had lower NTCP for liver, while the NTCP for lung from almost all patients was zero (Table 3). Significant NTCP for liver from patients of Group S‐I was observed (median value: 0.0000 vs 0.0027 %; *P* = 0.0208). NTCP for heart and lung was comparable (Table 4).

**Table 3:** Normal tissue complication probability (NTCP) results for heart, lung, and liver.

Group S‐I				Group R‐L			
Patient #	Pericarditis %	Liver failure %	Pneumonitis %	Patient #	Pericarditis %	Liver failure %	Pneumonitis %
1	0.0002	0.0000	0.0000	1	0.0039	0.0351	0.0004
2	0.0029	0.0000	0.0000	2	0.0046	0.0009	0.0000
3	0.0002	0.0013	0.0000	3	0.0027	0.0043	0.0000
4	0.0000	0.0000	0.0000	4	0.0006	0.0000	0.0000
5	0.0001	0.0000	0.0000	5	0.0072	0.0050	0.0000
6	0.0007	0.0001	0.0000	6	0.0032	0.0000	0.0000
7	0.0650	0.0014	0.0000	7	0.0095	0.0857	0.0000
8	0.0002	0.0000	0.0000	8	0.0003	0.0011	0.0000
9	0.0173	0.0000	0.0000	9	0.0050	0.0266	0.0000
10	0.0008	0.0001	0.0000	10	0.0001	0.0000	0.0000

**Table 4:** Statistical comparison of normal tissue complication probability (NTCP) results for heart, lung, and liver.

Endpoint	Group S‐I Median (range)	Group R‐L Median (range)	*P*‐value^a^
Pericarditis	0.0004 (0.0000,0.0029)	0.0036 (0.0001,0.0095)	0.5748
Liver failure	0.0000 (0.0000,0.0001)	0.0027 (0.0000,0.0351)	0.0208
Pneumonitis	0.0000 (0.0000,0.0000)	0.0000 (0.0000,0.0000)	0.3173

^a^ The Wilcoxon signed‐rank test was used.

## 4 DISCUSSION

As the first group to do so, in this paper, we evaluated the impacts of two different beam angle configurations in the IMPT treatment planning for distal esophageal carcinoma. The comparisons were carried out between the groups of S‐I oblique posterior versus R‐L oblique posterior beams. The assessments of plan quality in the nominal scenario and plan robustness with 13 uncertainty scenarios were carried out on the averaged 4D‐CT, the maximum inhalation phase, and the maximum exhalation phase, respectively. At last, interplay effect evaluation using the 4D dynamic dose calculation was performed.

For all patients, Orfit board and thermoplastic masks (Orfit, Wijnegem, Belgium) were used for immobilization to mitigate the setup variations. Different from other proton machines in the market, our treatment gantries are only half rotating. Therefore, there is no difference in the setup residual errors for the two different treatment angles since both involve couch and gantry rotation. However, it is true that for proton machines with full rotation, only gantry rotation is involved in the R‐L oblique posterior beam angle setting, while both couch and gantry rotation are involved in the S‐I oblique posterior beam angle setting. The latter might introduce additional setup residual errors in the treatment of distal esophageal carcinoma.

The idea of altering the IMPT beam angle from posterior R‐L oblique to posterior S‐I oblique pairs was initially motivated by the hypothesis‐generating inquiry that S‐I oblique posterior beams would better spare adjacent OARs located laterally to the target and have better plan robustness by going along with the S‐I axis that usually has the largest amount of respiratory motion. Generally speaking, on one hand, the R‐L oblique posterior beams have more potential to penetrate the nearby OARs, such as kidneys, lungs, liver etc., while the S‐I oblique posterior beams would have less beam paths through the diaphragm radially. On the other hand, the S‐I oblique posterior beams may traverse more volume of the spinal cord, thus possibly resulting in higher dose to this OAR. With the expected increased but clinically acceptable maximum spinal cord dose, the possible benefits of better OAR sparing and better plan robustness to respiratory motion make the evaluation of S‐I oblique posterior beam angles worthwhile—particularly for anatomically midline and posterior targets, such as distal esophageal cancer and pancreatic tumors, which can be affected by respiratory motion. Our findings confirmed this hypothesis, as reasonable amounts of potentially beneficial reductions of radiation dose in lungs, liver, and heart were observed in Group S‐I, with a slightly higher—but clinically acceptable—spinal cord maximum dose. It is important to note that all plans in Group S‐I still met our institutional dose‐volume constraints for spinal cord. Clinically, we observed no spinal cord toxicity as a result of this approach to date.^65^ Additionally, even though the slight worse hot spot control in target volume from Group S‐I was observed, we believe this had little influence for the plan for two reasons. First, the difference was quite small, <2%, and evidently under clinically acceptable level. Second, during respiratory motion, the most hot spot in a plan would mostly stay within the target volume, or occasionally move to one of the adjacent organs: lung, liver, and heart. All these organs are parallel‐type organs that have relatively large volume effect, especially for lung. Thus, the dose‐response may be closer to mean dose rather than the hot spot. In fact, comparable or even better mean dose results in these organs were observed in Group S‐I, which could be confirmed by the comparable or even better NTCP results.

As for plan robustness, using different beam angles did not appear to produce any adverse impact with the Group S‐I approach. This is understandable because identical optimization methods were applied. Group S‐I had even better plan robustness with regard to lung protection. The better plan robustness for lung from Group S‐I was expected as the new beam angles would traverse less lung volumes, and better focus proton delivery to the posterior mediastinal areas where the targets were located. Comparatively, Group R‐L beams would pass through a larger amount of lung tissue in the transverse dimension. Therefore, any misalignment or anatomical change in patients would have a larger impact on Group R‐L patients, which was demonstrated by our data. With regard to interplay effect considered, the better sparing of lung, liver, and heart in the Group S‐I patients demonstrated in our study could be attributed to the comprehension that the S‐I obliquely placed posterior beams would have better insensitivity to respiratory motion. Thus, clinically it should be chosen routinely for IMPT planning. At our institution, S‐I posterior oblique beams are now the preferred beam angle choice for distal esophageal cancer patients treated with IMPT.

This study has certain limitations. First, the patient groups chosen were heterogeneous and small in number, and also there may exist other characteristics that could bias the results. To help mitigate this effect, best efforts were given to match the CTV sizes and respiratory motion amplitude, so that the two groups became comparable both anatomically and also clinically, on the consensus that all patients had distal esophageal cancers requiring IMPT treatments. Second, our clinical experience as a new proton center is still relatively small^65^; we await more cancer patients’ proton experience in the future so we could help validate this experience with a larger patient cohort. Third, proton beam distal effects on OARs were not considered in this study. High linear energy transfer (LET) may appear in the distal fall‐off regions of a proton beam and may cause unexpected AEs to nearby OARs. At our institution, every plan will receive a second dose and LET calculation^66^, ^67^, ^68^ after the plan is generated by our TPS (Eclipse^TM^). The physicists and the attending physician will then check for overlap of high dose (at least 50% of the prescription dose) and high LET (at least 6 keV/µm) in nearby OARs during the LET‐guided plan evaluation. If high overlap is observed, the plan will be adjusted to minimize the overlap in nearby OARs. Finally, we caution that this experience should not be generically applied to patients in the setting of re‐irradiation, where spinal cord dose is often the limiting factor in terms of what is achievable. Our ultimate recommendation of using an S‐I oblique posterior beam arrangement as the baseline approach should always be based on the recognition that cases should still be evaluated on an individual basis.

## 5 CONCLUSIONS

When compared to the IMPT plans with R‐L oblique posterior beam arrangement, IMPT plans with S‐I oblique posterior beams had better sparing of liver, heart, and lungs, whereas resulted in a slight increase in the maximum dose of the spinal cord. Both sets of beam angle configurations resulted in clinically acceptable plan robustness. Moreover, S‐I oblique posterior beam angles led to more interplay‐effect resilient IMPT plans for distal esophageal cancer treatment. This study supports our choice of clinically using the S‐I posterior oblique beams, instead of R‐L posterior oblique beams, as our preferred method for the IMPT treatment of patients with distal esophageal carcinoma requiring chemoradiation.

## CONFLICT OF INTEREST

Terence T. Sio, MD, MS, provides strategic and scientific recommendations as a member of the Advisory Board and speaker for Novocure, Inc. This position has no relation to this manuscript. All other authors have no additional conflict to disclose.

## ETHICAL CONSIDERATIONS

This research was approved by the Mayo Clinic Arizona institutional review board (IRB, 13‐005709). The informed consent was waived by IRB protocol. Only image and dose‐volume data were used in this study. All patient‐related health information was removed prior to the analysis and also publication of the study.

## Supporting information


**Table S1**. Details of Beam Angle (G for gantry, T for table), IMPT machine characteristics, and beam energy information.
**Table S2**. Details of tumor motion amplitude in three directions.
