# Supplementary material for: Beam angle comparison for distal esophageal carcinoma patients treated with intensity‐modulated proton therapy
Source: J Appl Clin Med Phys. 2020 Oct 15;21(11):141–52. doi: 10.1002/acm2.13049 (PMC7700921; doi:10.1002/acm2.13049)
Supplement: Supplementary file 1 — Table S1. Details of Beam Angle (G for gantry, T for table), IMPT machine characteristics, and beam energy information. Table S2. Details of tumor motion amplitude in three directions. [file ACM2-21-141-s001.docx]

| Group S-I | | | Group R-L | | |  |
| --- | --- | --- | --- | --- | --- | --- |
| Patient # | Beam angles | Energy range (MeV) | Patient # | Beam angles | Energy range (MeV) | |
| 1 | T270, G160 | 100.5~205.3 | 1 | T0, G150 | 99.2~193.6 | |
|  | T270, G185 | 106.4~193.6 |  | T180, G150 | 107.8~203.1 | |
| 2 | T270, G155 | 83.7~195.9 | 2 | T0, G150 | 94.8~173.6 | |
|  | T270, G185 | 83.7~179.5 |  | T180, G150 | 90.1~177.5 | |
| 3 | T270, G155 | 113.2~191.3 | 3 | T0, G150 | 93.7~189.0 | |
|  | T270, G185 | 109.1~181.4 |  | T180, G150 | 93.2~200.4 | |
| 4 | T270, G155 | 100.7~193.6 | 4 | T180, G185 | 91.7~179.5 | |
|  | T270, G185 | 94.8~183.8 |  | T180, G150 | 93.2~191.3 | |
| 5 | T270, G160 | 110.5~191.3 | 5 | T0, G160 | 85.3~167.6 | |
|  | T270, G185 | 106.4~185.2 |  | T180, G160 | 88.5~167.6 | |
| 6 | T270, G160 | 85.3~187.1 | 6 | T0, G155 | 96.3~171.6 | |
|  | T270, G185 | 75~175.6 |  | T180, G160 | 100.7~181.4 | |
| 7 | T270, G160 | 76.8~161.5 | 7 | T0, G150 | 80.3~171.6 | |
|  | T270, G185 | 73.2~156.7 |  | T180, G145 | 93.2~191.3 | |
|  |  |  |  | T180, G10 | 71.3~153.5 | |
| 8 | T270, G160 | 97.8~189.0 | 8 | T180, G160 | 100.7~195.9 | |
|  | T270, G185 | 96.3~179.5 |  | T180, G180 | 96.3~179.5 | |
|  |  |  |  | T180, G40 | 137.7~203.1 | |
| 9 | T270, G150 | 91.7~181.4 | 9 | T0, G160 | 78.5~171.6 | |
|  | T270, G185 | 85.3~165.6 |  | T180, G160 | 85.3~175.6 | |
|  |  |  |  | T180, G25 | 82.0~155.1 | |
|  |  |  |  | T180, G90 | 132.1~179.5 | |
| 10 | T270, G160 | 96.8~189.0 | 10 | T0, G150 | 107.8~200.4 | |
|  | T270, G185 | 94.8~183.8 |  | T180, G150 | 105.0~205.3 | |

Supplemental Table 1. Details of Beam Angle (G for gantry, T for table), IMPT machine characteristics, and beam energy information.

Supplemental Table 2. Details of Tumor Motion Amplitude in Three Directions.

| Group S-I | | | | Group R-L | | | |
| --- | --- | --- | --- | --- | --- | --- | --- |
| Patient  # | S-I  (cm) | A-P  (cm) | R-L  (cm) | Patient  # | S-I  (cm) | A-P  (cm) | R-L  (cm) |
| 1 | 0.8 | 0.3 | 0.2 | 1 | 1.0 | 0.3 | 0.3 |
| 2 | 0.8 | 0.9 | 0.9 | 2 | 0.8 | <0.2 | 0.3 |
| 3 | 0.9 | <0.2 | 0.9 | 3 | 0.7 | 0.4 | 0.4 |
| 4 | 0.8 | 0.9 | 0.3 | 4 | 0.8 | <0.2 | 0.6 |
| 5 | 0.6 | 0.2 | <0.2 | 5 | 1.0 | 0.5 | <0.2 |
| 6 | 0.7 | 0.6 | <0.2 | 6 | 0.9 | 0.5 | <0.2 |
| 7 | 0.8 | 0.4 | 0.3 | 7 | 0.8 | 0.4 | 0.3 |
| 8 | 0.7 | <0.2 | 0.4 | 8 | 1.0 | 1.0 | <0.2 |
| 9 | 0.5 | <0.2 | <0.2 | 9 | 0.6 | 0.2 | 0.2 |
| 10 | 0.6 | 0.4 | 0.3 | 10 | 0.6 | 0.3 | <0.3 |

In Group R-L, the largest tumor motion is always in the S-I direction, while in Group S-I there are two exceptions: Patient 2 and 4. However, the S-I tumor motions of patient 2 and 4 are also comparably large (0.8 vs 0.9cm), which is still consistent with our investigation foundation that the S-I axis usually has the largest amount of respiratory motion.
